# 90232 Implementing the innovative academic Learning Health System Scholars (aLHSS) Postdoctoral Training Program (TL1) at Wake Forest University Health Sciences (WFUHS)

**DOI:** 10.1017/cts.2021.739

**Published:** 2021-03-30

**Authors:** Rachel Woodside, Gary Rosenthal, Claudia Olivier

**Affiliations:** Wake Forest Clinical and Translational Science Institute

## Abstract

ABSTRACT IMPACT: Learning Health System (LHS) Science that trains postdoctoral scholars from diverse professional backgrounds in methodological and professional skills to implement rigorous research in health care systems and populations, and to disseminate the findings of such research to improve healthcare delivery OBJECTIVES/GOALS: The WFUHS CTSA developed an innovative TL1 in Learning Health System (LHS) Science that trains postdoctoral scholars from diverse professional backgrounds in methodological and professional skills to implement rigorous research in health care systems and populations, and to disseminate the findings of such research to improve healthcare delivery METHODS/STUDY POPULATION: Training is centered around formal LHS science coursework and mentored research projects that address a pressing health system issue. Projects are closely guided by a primary mentor and a multidisciplinary mentoring team. Program mission and competencies were carefully evaluated in a competency-course matrix to design new courses for the LHS Certificate and MS program in Translational and Health System Science (THSS). Course domains include biomedical informatics; improvement and implementation science; system science and organizational change management; stakeholder engagement, leadership, and research management; ethics of health systems research; and health systems research methods. Scholars set up Individual Development Plans (IDP) and self-assess 7 domains of LHS core competencies. RESULTS/ANTICIPATED RESULTS: The first professionally diverse group of scholars (MD, PhD, DrPH, PharmD) began the program in Summer 2020; onboarding was conducted virtually. Scholars currently conduct most of their research and training in a virtual, synchronous format. Each developed a detailed IDP and LHS research project, which was reviewed by their LHS mentoring teams (includes a primary mentor, co-mentor, TL1 core faculty mentor, peer mentor, and health system mentor). Coursework, leading to a 1-year certificate or 2-year MS degree, was selected based on individual background and career goals and was begun in August 2020. In addition to the courses noted above, Scholars are embedded in a healthcare improvement team. We use the process of a LHS and hold weekly TL1 leadership meetings to swiftly address challenges and implement improvements DISCUSSION/SIGNIFICANCE OF FINDINGS: We envision that TL1 Scholars will build independent LHS research programs or lead health system innovation. Program evaluation includes assessments of Scholar fluency in LHS competencies and attainment of key milestones during and after training. Annual TL1 faculty retreats will address program fidelity and implementation of program refinements

